# Release of Propolis Phenolic Acids from Semisolid Formulations and Their Penetration into the Human Skin *In Vitro*

**DOI:** 10.1155/2013/958717

**Published:** 2013-05-23

**Authors:** Modestas Žilius, Kristina Ramanauskienė, Vitalis Briedis

**Affiliations:** Department of Clinical Pharmacy, Lithuanian University of Health Sciences, A. Mickevičius Street 9, 44307 Kaunas, Lithuania

## Abstract

Antioxidant and free radical scavenging effects are attributed to phenolic compounds present in propolis, and when delivered to the skin surface and following penetration into epidermis and dermis, they can contribute to skin protection from damaging action of free radicals that are formed under UV and premature skin aging. This study was designed to determine the penetration of phenolic acids and vanillin into the human skin *in vitro* from experimentally designed vehicles. Results of the study demonstrated the ability of propolis phenolic acids (vanillic, coumaric, caffeic, and ferulic acids) and vanillin to penetrate into skin epidermis and dermis. The rate of penetration and distribution is affected both by physicochemical characteristics of active substances and physical structure and chemical composition of semisolid vehicle. Vanillin and vanillic acid demonstrated relatively high penetration through epidermis into dermis where these compounds were concentrated, coumaric and ferulic acids were uniformly distributed between epidermis and dermis, and caffeic acid slowly penetrated into epidermis and was not determined in dermis. Further studies are deemed relevant for the development of semisolid topically applied systems designed for efficient delivery of propolis antioxidants into the skin.

## 1. Introduction

Human skin protects an organism from constant exposure to physical, chemical, biological, and environmental factors [1]. Ultraviolet (UV) radiation is one of the major causes of formation of reactive oxygen species (ROS) and radicals, which are associated with UV-induced skin damages: skin aging, cancer, and other skin diseases [2, 3]. Delivery of external antioxidant molecules to the sites of potential UV-induced formation of ROS should be considered when protecting the skin from oxidative damage and retarding aging-related processes. Antioxidant, free radical scavenging, anti-inflammatory, antitumoral, antimicrobial, and wound healing properties have been confirmed for propolis and its products both *in vitro* and *in vivo* studies, and these attributes are linked with the presence of phenolic compounds, including flavonoids, phenolic acids, and their esters [4–8]. A contribution of propolis phenolic compounds to the skin protection is not widely established. Recently published data demonstrate the ability of propolis extract to prevent UV irradiation-induced oxidative stress in the skin [5]. Strong antioxidant and free radical scavenging properties of propolis are conditioned by presence of phenolic acids (rosmarinic, chlorogenic, caffeic, and ferulic acids) and phenolic aldehyde vanillin [9]. Brazilian researches have evaluated the propolis extract release from topical formulations and the permeation in skin pig ear and in the whole hairless mice skin [10–12]. Also the potential applicability of Brazilian propolis extracts for the prevention of oxidative stress in the skin due to UV irradiation was studied. To cultivate a more complete understanding of the protective capabilities of propolis extracts, topical formulations that diffuse more effectively through the skin could be developed and additional studies be performed.

Therefore, the objective of this study was to evaluate the release of propolis phenolic acids and vanillin from experimental semisolid formulations and their penetration into the human skin layers *in vitro*. The current study was designed to evaluate the ability of propolis phenolic acids and phenolic aldehyde vanillin to penetrate human skin from different carriers that were designed and developed for topical application.

## 2. Materials and Methods

### 2.1. Chemicals and Reagents

Raw propolis was obtained from UAB Medicata Filia (Vilnius, Lithuania), and soft propolis extract was obtained from UAB Valentis (Kaunas, Lithuania); acetonitrile (Chromasolv) was gradient grade for HPLC, ≥99.9% and acetic acid (glacial), ≥99.8% pure quality (Sigma-Aldrich Chemie GmbH, Steinheim, Germany). The standards of phenolic compounds for HPLC were purchased from Sigma-Aldrich Chemie GmbH (Steinheim, Germany): vanillic acid (≥98%), caffeic acid (≥98%), vanillin (≥99%), coumaric acid (≥98%), and ferulic acid (≥99%). Purified water for preparation of solvents was filtered through the Millipore HPLC grade water preparation cartridge (Millipore, Bedford, PA, USA). Vaseline (white), glycerol, span 80 (sorbitan oleate), poloxamer 407 (polyoxyethylene-polyoxypropilene-polyoxyethylene tri-block copolymer), and sodium carboxymethylcellulose (50–200 cP) were purchased from Sigma-Aldrich Chemie GmbH (Steinheim, Germany); lanolin, anhydrous (CPC W. M. GmbH, Germany); Pionier PLW (mixture of mineral oil and polyethylene) (Hansen & Rosenthal, Hamburg, Germany); sodium chloride (Carl Roth GmbH, Karlsruhe, Germany) and sodium azide (POCh, Gliwice, Poland).

### 2.2. Aqueous and Ethanolic Propolis Extracts

Propolis sample-to-solvent ratio was 1 : 10 (w/v) when preparing aqueous propolis extract. Phenolic compounds from raw propolis were extracted using purified water with stirring at 70°C for 1 hour using a hotplate magnetic stirrer IKAMAG C-MAG HS7 (IKA-Werke GmbH & Co. KG, Staufen, Germany) [13, 14]. Produced aqueous propolis extract was cooled to room temperature and filtered.

### 2.3. Propolis Extracts Analysis by High-Performance Liquid Chromatography

Four phenolic acids (coumaric, ferulic, caffeic, and vanillic acids) and vanillin were identified in propolis extracts using Agilent 1260 Infinity capillary LC (Agilent Technologies, Inc., Santa Clara, CA, USA) with Agilent diode array detector (DAD) and applying validated HPLC method for quantification of the above-mentioned phenolic compounds. The separation of phenolic acids and vanillin was achieved with an ACE C18 column (150 × 0.5 mm, 5 *μ*m particle size) using the mobile phase composed of acetonitrile (solvent A) and 0.5% (v/v) acetic acid in water (solvent B). The linear elution gradient from 1 to 21% of solvent A in B 25 min was applied. The injection volume was 0.2 *μ*L, the flow rate was 20 *μ*L/min, and the column temperature was 25°C. The specificity and identification of phenolic acids were confirmed with a PDA detector and UV spectra of the appropriate standards in the wavelength range of 210–600 nm. The integration of phenolic acids peaks was performed at 290 nm [15].

All propolis samples were filtered through 0.20 *μ*m sterile nylon membrane filters (diameter of 25 mm) for syringes (Sartorius Stedim Biotech GmbH, Goettingen, Germany) prior to performing HPLC analysis.

### 2.4. Formulation of Semisolid Preparations with Propolis Extracts

Choice of excipients for ointment and w/o cream semisolid vehicles were developed with the reference to earlier published research data [16]. The components of semisolid propolis preparations are listed in Table 1. The compositions of the semisolid vehicles were optimized applying the experimental central composite design model. Optimization was performed referring to the released quantity of total phenolic compounds after 6 hours.

The experimental propolis ointment and water-in-oil (w/o) cream were prepared at room temperature using the Unguator 2100 mixing machine (GAKO International GmbH, Munich, Germany). Ointment base components were mixed until homogeneity. Later soft propolis extract was added and dispersed in the ointment base. The final mixing step in propolis ointment formulation was performed for 2 min using normal general mixing procedure. Formulation of propolis w/o cream was performed by addition of aqueous phase to homogeneous oily phase containing emulsifying agent to produce primary emulsion. Soft propolis extract was added for dispersion, and final mixing was performed at mixing under 750 rpm, oscillation 1500 rpm, and continued for 2.25 min. Experimental propolis hydrogel formulation was prepared by cold method [17].

### 2.5. The Viscosity Determination of Semisolid Propolis Preparations

Dynamic viscosity (Pa·s) of experimental semisolid propolis preparations (Table 1) was assessed at 32°C temperature using rotary viscometer ST-2010 (JP Selecta S.A., Spain). Cylindrical spindle (R4) rotating speed was 10.0 rpm. The duration of each measurement was 10 s (*n* = 3).

### 2.6. *In Vitro* Release Study

*In vitro* release experiments were performed (*n* = 3) using the modified Franz type diffusion cells and the dialysis membranes of natural cellulose Cuprophan (Medicell International Ltd., London, UK) [11, 18]. A diffusion area was 1.77 cm^2^. The infinite dose of the donor phase was placed into the diffusion cell. The aqueous receptor medium was stirred using the hotplate magnetic stirrer IKAMAG C-MAG HS7 (IKA-Werke GmbH & Co.KG, Staufen, Germany) maintaining the temperature of 32 ± 0.1°C. The samples from the receptor solution were removed at 0.5, 1, 2, 4, 6, 8 hour and replaced with the same volume of fresh receptor solution. All samples were analyzed by HPLC. 

### 2.7. *In Vitro* Skin Permeation Study

Caucasian women (age range 25–40 years) abdominal skin was obtained from Department of Plastic and Reconstructive Surgery, Hospital of Lithuanian University of Health Sciences, after cosmetic surgery, and was stored at −20°C not longer than 6 months before use. Kaunas Region Bioethical Committee has approved the use of human skin for transdermal penetration studies. *In vitro* skin permeation experiments (*n* = 6) were performed using Bronaugh type flow-through diffusion cells with full-thickness human skin. Acceptor phase was circulated by Masterflex L/S peristaltic pump with multichannel pump head (Cole-Parmer Instrument Co., IL, USA) [19, 20]. The efficient diffusion area in the cells was 0.64 cm^2^. The diffusion cells with human skin samples were placed on the heating block maintaining 37°C by a Grant GD120 thermostated circulating water bath (Grant Instruments Ltd., Cambridge, Great Britain).

The equilibration of the diffusion testing system was performed for 12 hours, during which the circulation of 0.9% NaCl solution containing 0.005% NaN_3_ was maintained underneath the skin samples. After the equilibration period, the infinite dose of the donor phase was applied on the outer human skin side (*stratum corneum* side) surface, and the diffusion cells were covered with aluminum foil. Circulation rate of the acceptor medium underneath the skin samples (0.9% NaCl solution with 0.005% NaN_3_) was 0.6 mL/min.

The donor phase was removed from the human skin surface after 24 hours, and *stratum corneum* side was rinsed 2 times with 96.3% ethanol and 3 times with 0.9% NaCl. The skin samples (0.64 cm^2^) were trimmed off removing the outer residuals. Epidermis was separated from dermis applying dry heat separation method. The skin sample (*epidermis* side) was placed on the hot (approx. 60°C) metal surface for short time (1-2 s), and epidermis was peeled off. Then, epidermis and dermis were separately extracted with 1 mL of a mixture of methanol and deionized water (1 : 1) for 30 min under sonication in a Bandelin Sonorex Digitec bath (DT 156, Bandelin electronic GmbH & Co. KG, Berlin, Germany).

### 2.8. Statistical Analysis

Experimental optimization was performed applying surface response central composite design in Design-Expert 6.0.8 software. Statistical analysis of experimental data was performed using SPSS software (version 19.0). Mann-Whitney *U* test and one-way ANOVA (Tukey's Honestly Significant Difference criterion) were used for data analysis. Correlation analysis was performed applying Spearman's rank coefficient.

## 3. Results and Discussion

**Figure 1:**
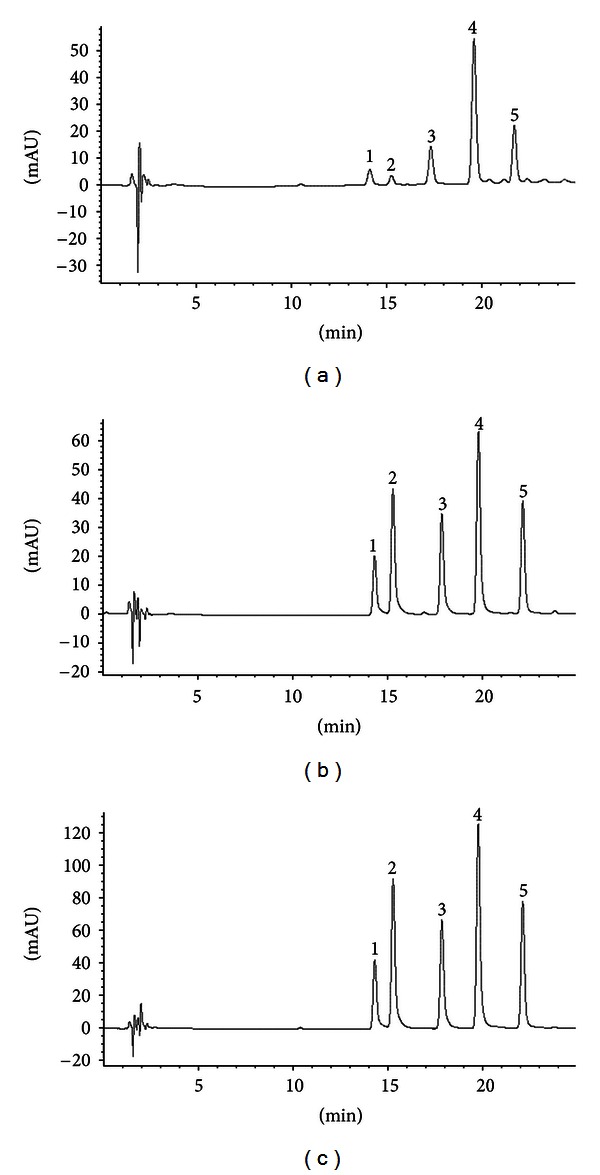
Chromatographic determination of vanillic acid (1), caffeic acid (2), vanillin (3), coumaric acid (4), and ferulic acid (5) in aqueous propolis extract (a), epidermis (b), and dermis (c).

Phenolic acids and phenolic aldehide vanillin were identified and quantitatively assessed in the aqueous and soft propolis extracts during experimental studies (Table 2). The characteristic chromatograms of vanillic, caffeic, coumaric, ferulic acid, and vanillin in propolis aqueous extract, epidermis, and dermis are presented in Figure 1. Validated chromatographic separation method ensured efficient separation and quantification of phenolic compounds in propolis extracts and specimens of skin epidermis and dermis. No external interference with the accuracy and separation was identified when analyzing skin specimens. The results of the analysis supported published data [15, 21], confirming that coumaric, ferulic acids, and vanillin are the dominating components in raw Lithuanian propolis (Table 2). Aqueous propolis extract was used as an active substance's form, for development of hydrophilic gel systems for further studies (Table 1). Meanwhile, soft propolis extract was used as an active substance's form in formulation of hydrophobic and emulsion systems (Table 1).

**Figure 2:**
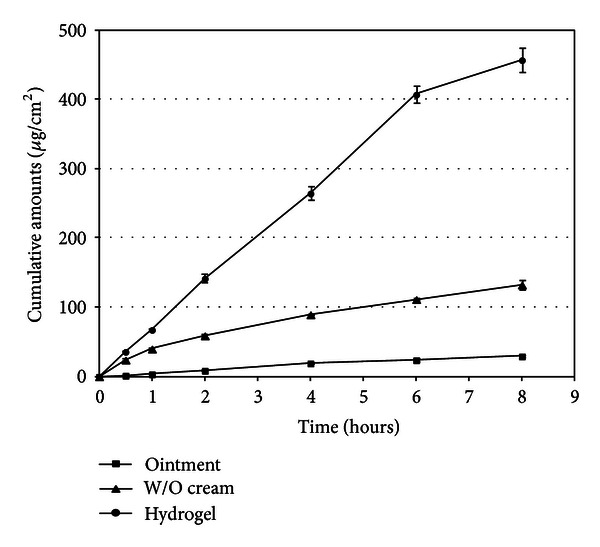
Release profiles of cumulative amounts of vanillic, caffeic, coumaric, ferulic acids, and vanillin from ointment, w/o cream, and hydrogel.

The results of the phenolic compounds release studies were in line with the published data, demonstrating that the release of drug substances from semisolid vehicles is influenced not only by the properties of drug molecule but also by the properties of the vehicle and concentration of the drug in the preparation [22]. Release data demonstrated that after 8 hours hydrophilic gel released nearly the whole amount of phenolic compounds, and only up to 5% and 22% of phenolic compounds were released from ointment and w/o cream, respectively (Figure 2). Evaluating the released quantities of each phenolic compound from ointment and w/o cream, the similarity of the profiles was evident. Comparison of the released fractions demonstrated the domination of vanillin (39%) and vanillic acid (32%) in case of w/o cream, and in case of ointment vanillic acid (16%) was prevailing after 8 hours. No traces of caffeic acid were determined when the evaluation of the phenolic compounds from ointment has been performed, and only 9% of caffeic acid (lowest amount amongst evaluated phenolic compounds) was released from w/o cream. Nearly complete release of phenolic acids and vanillin from hydrogel can be attributed to possible gel dissolution after penetration of aqueous acceptor medium (Figure 2). Observed release profiles of phenolic acids and vanillin could be related to lipophilicity of the tested substances and semisolid vehicle. Phenolic acids are partly lipophilic by nature. Average calculated log⁡⁡*P* value (ALOGPS 2.1) is 1.18 for vanillin, 1.24 for vanillic acid, 1.25 for caffeic acid, 1.42 for ferulic acid, and 1.53 for coumaric acid. Appealing to calculated log⁡⁡*P* values, it could be concluded that vanillin and vanillic acid are less lipophilic if compared to ferulic and coumaric acids. Referring to the experimental data it could be concluded that active compounds less affined to semisolid vehicle should be released first. Vehicles with lower content of lipophilic substances (e.g., w/o cream compared to ointment) released phenolic compounds more intensively, especially the more hydrophilic compounds, and this conforms to the published data [11]. Meanwhile the ointments with higher content of lipophilic substances restrained the release of more lipophilic compounds into aqueous receptor medium (“like dissolves like”). 

**Figure 3:**
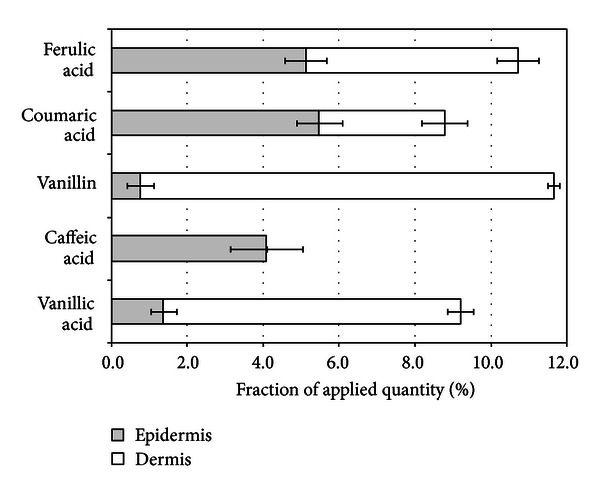
Penetration of ferulic, coumaric, caffeic, vanillic acids, and vanillin into epidermis and dermis after aqueous propolis extract application for 24 hours.

Aqueous propolis extract was applied to the human skin *in vitro* to evaluate the ability of vanillin, and vanillic, caffeic, coumaric, and ferulic acids to penetrate into/through epidermis and dermis. Evaluation of penetrated fraction of phenolic acids demonstrated that after 24 hours epidermis contained approx. 5% of coumaric and ferulic acids, and in dermis 4% of coumaric acid and 6% of ferulic acid were determined (Figure 3). Vanillin and vanillic acid relatively easily penetrated into epidermis and similarly easily, thus the determined contents of these compounds were low in epidermis (1-2%) and higher in dermis (11% and 8%, resp.). The fraction of caffeic acid in epidermis reached 4%, and it was not determined in dermis. Such differences in penetration results could be related to lipophilicity of phenolic compounds. Evaluation of Spearmen's rank correlation coefficients demonstrated the existing statistically significant (*P* < 0.01) direct correlation (*R* ^2^ = 0.904) between lipophilicity of compounds (log⁡⁡*P* value) and their ability to penetrate into epidermis, whereas indirect correlation (*R* ^2^ = −0.965) was statistically significant (*P* < 0.01) when lipophilicity of compounds was related to their ability to penetrate into dermis. It could be concluded that more lipophilic coumaric, ferulic, and caffeic acids were predominantly concentrated in epidermis, and only limited quantities of coumaric and ferulic acids penetrated into dermis. Meanwhile less lipophilic vanillin and vanillic acid were permeating epidermis and penetrating into dermis. These results indicate the dependence of phenolic compounds distribution profile on their lipophilicity and properties of skin layers.

**Figure 4:**
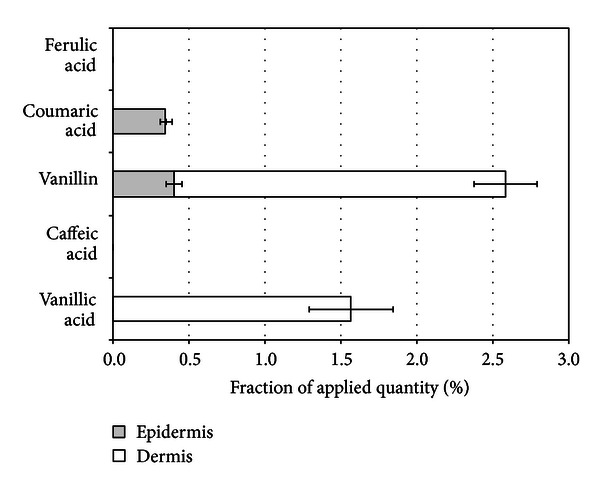
Penetration of coumaric, vanillic acids, and vanillin into epidermis and dermis after propolis aqueous extract containing hydrogel application for 24 hours.

The quantities of phenolic acids and vanillin were statistically significantly lower (*P* < 0.05) both in epidermis and dermis when hydrophilic gel was used as a vehicle for propolis phenolic compounds if compared to application of aqueous propolis extract (Figure 4). It should be noted that hydrophilic gel supported penetration of vanillin and vanillic acid into dermis, while coumaric acid was determined only in epidermis. Ferulic and caffeic acids were not determined in epidermis, though both these compounds were penetrating into the skin when aqueous propolis extract has been applied. Noteworthy to mention is that hydrophilic gel was nearly completely releasing all tested phenolic compounds in this study. The penetration results suggest the existence of complex interaction between semisolid vehicle and skin layers, and it could demonstrate critical effects on compound penetration into the skin.

**Figure 5:**
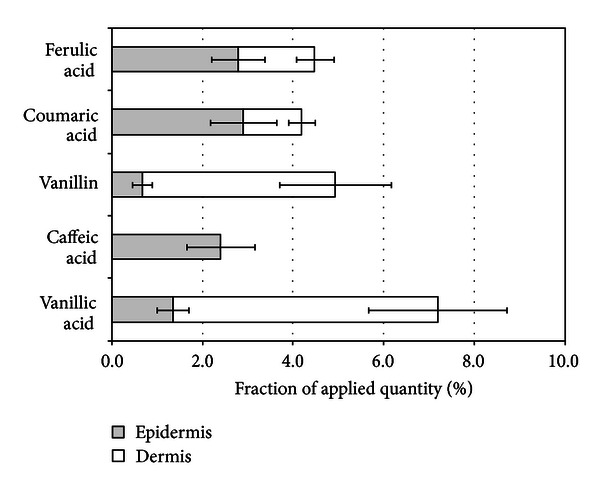
Penetration of ferulic, coumaric, caffeic, vanillic acids, and vanillin into epidermis and dermis after propolis cream application for 24 hours.

**Figure 6:**
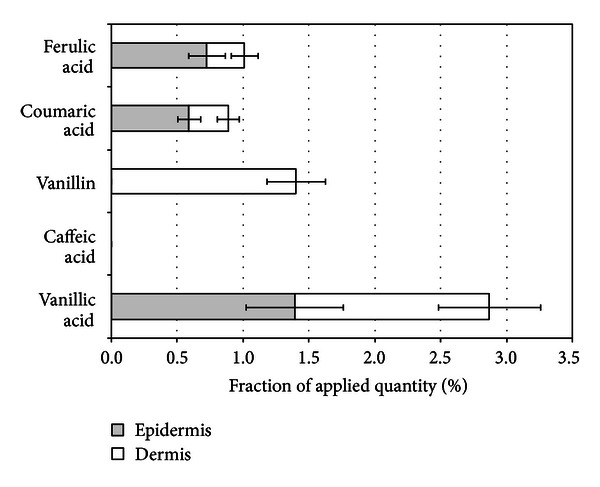
Penetration of ferulic, coumaric, vanillic acids, and vanillin into epidermis and dermis after propolis ointment application for 24 hours.

Comparative evaluation of hydrophobic and emulsion type vehicles regarding their effect on skin penetration behavior of propolis active phenolic constituents revealed higher penetration values when emulsion system was applied (Figure 5). Penetration profile of phenolic acids and vanillin from emulsion systems was similar to the penetration profile resulting from application of propolis aqueous extract, but the determined absolute quantities of phenolic compounds were approximately 2-fold lower. Data presented in Figures 5 and 6 demonstrate that penetration of coumaric and ferulic acids from w/o cream into skin was approximately 4-fold higher (*P* < 0.05) when compared to results after application of ointment. Vanillin and vanillic acid penetrated into dermis, but only in the case of vanilic acid significant difference (*P* < 0.05) between penetration results from cream and ointment into skin was confirmed. Both in the case of vanillin and vanillic acid epidermis did not present a barrier for penetration. Caffeic acid had no opportunity to enter epidermis as it was not released from the utilized ointment vehicle as was confirmed by release studies. When w/o cream vehicle was utilized, presence of caffeic acid was identified in epidermis, but it was not determined in dermis.

The observed differences of penetration of phenolic acids and vanillin after application of cream and ointment could be partially explained by respective differences in released amounts of phenolic acids and vanillin from cream and ointment. Statistical analysis (ANOVA) of penetration *in vitro* data confirmed the statistically significant (*P* < 0.05) difference of quantities of phenolic compounds in human skin after application of w/o cream compared to ointment or gel, thus confirming suitability of this w/o emulsion system for delivery of propolis phenolic compounds.

Published scientific data demonstrate that penetration into the skin increases with the increasing hydration of skin tissue [23]. Pharmaceutical and cosmetic semisolid vehicles designed for topical application can increase skin hydration by limiting water evaporation from the skin surface or by supplying additional water amount to *stratum corneum* from the vehicle. Surface active agents employed as emulsifying, wetting, and solubility modifying excipients can negatively affect barrier function of the skin thus impacting on the penetration of external chemical substances into skin. It could be supposed that surface active agents present in emulsion type and hydrophobic systems have influenced the penetration of phenolic compounds into the skin through modification of skin barrier function. The data from this study showed that the highest permeation values of phenolic compounds have been inherent for w/o emulsion system, so emulsion's aqueous phase may have affected the penetration process. The results of the study demonstrated that the composition of the vehicles affects both the release of propolis active components and their distribution in the skin.

## 4. Conclusion

Study results indicate the suitability of developed vehicle systems for incorporation of propolis extracts. The release profiles of vanillic acid, caffeic acid, ferulic acid, coumaric acid, and vanillin were reproducible when optimized formulations were applied. Penetration and permeation of phenolic acids and vanillin into human skin layers *in vitro* were confirmed. Direct correlation between lipophilicity of compounds and their ability to penetrate into epidermis was demonstrated, whereas indirect correlation was established when lipophilicity of compounds was related to their ability to penetrate into dermis. The observed differences of penetration of phenolic compounds could be attributed to specific characteristics of semisolid vehicle. Gelification of propolis aqueous extract resulted in significant modification of qualitative and quantitative profile of phenolic acids and vanillin in epidermis and derma, thus suggesting the active role of the hydrophilic vehicle in penetration/permeation process. Most efficient process of phenolic acids and vanillin penetration was determined when emulsion type disperse system was applied, and the quantities of individual compounds were a few fold higher if compared to ointment though penetration profile was similar. Further research and development of efficient semisolid topical formulations for achieving higher penetration of most efficient antioxidant components from propolis extracts are deemed relevant and require further studies.

## Figures and Tables

**Table 1:** The composition of semisolid propolis preparations after optimization.

Excipient	Ointment (%)	W/O cream (%)	Hydrogel (%)
Vaseline, white	68.4	—	—
Lanolin, anhydrous	13.6	—	—
Glycerol	15	—	—
Soft propolis extract	3	3	—
Pionier PLW	—	50	—
Span 80	—	7	—
Purified water	—	40	8.5
Poloxamer 407	—	—	20
Sodium carboxymethylcellulose	—	—	1.5
Aqueous propolis extract	—	—	70

Dynamic viscosity (Pa · s)	1.95 ± 0.10	2.68 ± 0.04	7.83 ± 0.05

**Table 2:** Quantitative profile of identified phenolic compounds in Lithuanian propolis extracts.

Solute	Vanillic acid	Caffeic acid	Vanillin	Coumaric acid	Ferulic acid
Aqueous extract (1 : 10) (mg/mL)	0.207 ± 0.012	0.050 ± 0.003	0.277 ± 0.011	0.516 ± 0.026	0.308 ± 0.015
Soft propolis extract (mg/g)	4.132 ± 0.023	1.432 ± 0.046	6.521 ± 0.011	18.469 ± 0.041	12.937 ± 0.011
